# The role of homeostatic regulation between tumor suppressor DAB2IP and oncogenic Skp2 in prostate cancer growth

**DOI:** 10.18632/oncotarget.2228

**Published:** 2014-07-17

**Authors:** Yuh-Shyan Tsai, Chen-Li Lai, Chih-Ho Lai, Kai-Hsiung Chang, Kaijie Wu, Shu-Fen Tseng, Ladan Fazli, Martin Gleave, Guanghua Xiao, Leah Gandee, Nima Sharifi, Loredana Moro, Tzong-Shin Tzai, Jer-Tsong Hsieh

**Affiliations:** ^1^ Department of Urology, Medical College and Hospital, National Cheng Kung University, Tainan, Taiwan; ^2^ Department of Urology, University of Texas Southwestern Medical Center, Dallas, TX, USA; ^3^ School of Medicine and Graduate Institute of Basic Medical Science, China Medical University, Taichung,Taiwan; ^4^ Department of Cancer Biology, Lerner Research Institute, Cleveland Clinic, Cleveland, OH, USA; ^5^ Department of Urology, The First Affiliated Hospital, Medical School of Xi'an Jiaotong University, Xi'a, China; ^6^ Department of Bioengineering, University of Texas at Arlington, Arlington, TX, USA; ^7^ Vancouver Prostate Center, University of British Columbia, Vancouver, British Columbia, Canada; ^8^ Department of Clinical Sciences, University of Texas Southwestern Medical Center, Dallas, TX, USA; ^9^ Institute of Biomembranes and Bioenergetics, National Research Council (C.N.R.), Bari, Italy; ^10^ Graduate Institute of Cancer Biology, China Medical University, Taichung, Taiwan

**Keywords:** prostate neoplasm, Skp2, DAB2IP, ubiquitin

## Abstract

Altered DAB2IP gene expression often detected in prostate cancer (PCa) is due to epigenetic silencing. In this study, we unveil a new mechanism leading to the loss of DAB2IP protein; an oncogenic S-phase kinase-associated protein-2 (Skp2) as E3 ubiquitin ligase plays a key regulator in DAB2IP degradation. In order to unveil the role of Skp2 in the turnover of DAB2IP protein, both prostate cell lines and prostate cancer specimens with a variety of molecular and cell biologic techniques were employed. We demonstrated that DAB2IP is regulated by Skp2-mediated proteasome degradation in the prostate cell lines. Further analyses identified the N-terminal DAB2IP containing the ubiquitination site. Immunohistochemical study exhibited an inverse correlation between DAB2IP and Skp2 protein expression in the prostate cancer tissue microarray. In contrast, DAB2IP can suppress Skp2 protein expression is mediated through Akt signaling. The reciprocal regulation between DAB2IP and Skp2 can impact on the growth of PCa cells. This reciprocal regulation between DAB2IP and Skp2 protein represents a unique homeostatic balance between tumor suppressor and oncoprotein in normal prostate epithelia, which is apparently altered in cancer cells. The outcome of this study has identified new potential targets for developing new therapeutic strategy for PCa.

## INTRODUCTION

Prostate cancer continues as the leading male malignancy with significant mortality in the United States [1]; for example, an estimated 233,000 new cases and 29,480 deaths in 2014 [1]. Several unique genetic events were reported to be associated with the development of prostate cancer, including NKX3.1 inactivation, TMPRSS2-ERG fusion, MYC amplification, PTEN mutation, and EZH2 overexpression [2]. In addition to genetic event, our data indicate that DAB2IP, a novel family of RasGTPase-activating protein family as a potent tumor suppressor, is epigenetically silenced [3, 4], which is suppressed by EZH2 and other epigenetic machinery such as DNA methylation and histone acetylation [5-7]. DAB2IP plays an important role in regulating the cell growth and survival of prostate cancer [4] through its GAP domain in suppressing Ras-Raf-ERK activation or proline-rich (PR) domain in suppressing PI3K-dependent Akt phosphorylation. Also, DAB2IP can elicit cell apoptosis via apoptosis-stimulated kinase (ASK1)-JNK pathway [8]. Furthermore, DAB2IP can prevent the progression of prostate cancer [5, 9] by inhibiting epithelial-to-mesenchymal transition (EMT) via Wnt-elicited β-catenin pathway.

S-phase-associated kinase protein-2 (Skp2) is a member of Skp, Cullin, F-box containing complex [10] that functions as an ubiquitin E3 ligase, which is significantly elevated in prostate cancer. A genomic analysis reported increased copy number of *Skp2* gene in advanced metastatic prostate cancer [11]. Skp2 can regulate several cellular functions responsible for prostate cancer progression, including cell cycle progress, signal transduction, or DNA repair [12]. Noticeably, these substrates includes p27 [13], p21 [14], BRCA2 [15], smad4 [16], and Myc [17]. On the other hand, several factors can influence Skp2 activity, stability, and subcellular translocation. Akt appears to a key factor to phosphorylate Skp2 and cause activated Skp2 translocation into cytoplasm, which also prevents Skp2 from degrading by anaphase-promoting complex/cyclosome-Cdh1 (APC/C-Cdh1) complex [18, 19]. It is known that Skp2 can be regulated by Wnt-signaling pathway in human invasive urothelial cancer cells through the binding of TCF4 and β-catenin to its promoter [20] or through NF-κB, p53 and Akt/GSK-3β pathway [21]. However, the regulation of Skp2 in prostate epithelia remained largely unknown. Therefore, these findings prompt us to explore the relationship between DAB2IP and Skp2 in human prostate epithelial and cancer cells.

## RESULTS

### Skp2-mediated ubiquitin-proteasome system (UPS) regulates post-translational expression of DAB2IP

**Figure 1:**
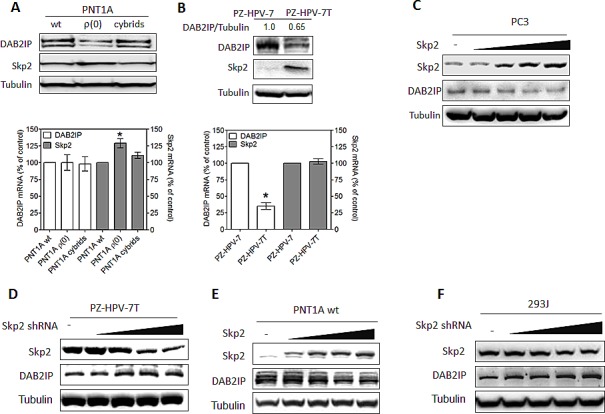
Inverse correlation between DAB2IP and Skp2 expression in prostatic cells (A) Both DAB2IP and Skp2 expression were analyzed using western blot (upper panel) and their mRNA expression were determined using qRT-PCR (lower panel) in PNT1A (wide type, wt), ρ(0) and Cybrids. Data are represented as mean ± SEM. (B) Both DAB2IP and Skp2 protein expression were analyzed using western blot (upper panel) and their mRNA expression determined using qRT-PCR (lower panel) in PZ-HPV-7 and PZ-HPV-7T cells. Data represented as mean ± SEM. (C-F) PC3, PZ-HPV-7T, PNT1A wt, and 293J cells were transfected with incremental concentration of different plasmids. Cell lysates were subjected to western blot probed with DAB2IP or Skp2 antibody. The intensity of each band was measured using the Imaging-Pro Plus and normalized with Tubulin.

We noticed that there is an inverse correlation between DAB2IP and Skp2 protein expression (Fig. 1A) in an immortalized normal prostate cell line (PNT1A) and its derivative PNT1A ρ(0), a mitochondrial DNA-deficient cell with neoplastic phenotypes [22]. Noticeably, DAB2IP protein levels in cybrids [22], derived from PNT1A ρ(0) after restoring mitochondrial DNA by fusing with platelets, were similar to those in PNT1A cells (Fig. 1A). No difference of DAB2IP mRNA levels in these cells was detected (Fig. 1A, lower panel), which rules out the transcriptional regulation. A similar expressions pattern of DAB2IP protein was also observed in another immortalized normal prostate cell line (PZ-HPV-7) and its tumorigenic subline (PZ-HPV-7T) [23] (Fig. 1B). By manipulating Skp2 expression level using cDNA or shRNA transfection in PC3, PNT1A, PZ-HPV-7T and 293J cells, the inverse correlation of DAB2IP and Skp2 protein expression was observed (Fig. 1C-F).

**Figure 2:**
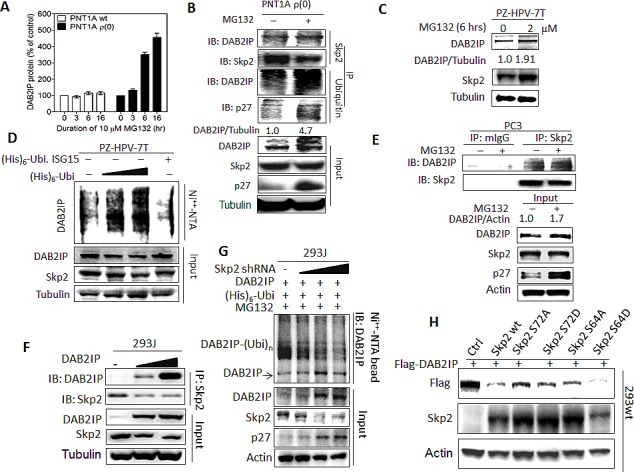
Regulation of DAB2IP expression by Skp2 mediated UPS (A) PNT1A wt, and ρ(0) cells were treated with MG132 (10 μM) for the indicated duration, and the DAB2IP expression was analyzed using western assays The density of bands was measured using the Image-Pro plus and normalized with Tubulin. The fold change [38] was calculated. Data are represented as mean ± SEM. (B) PNT1A ρ(0) cells were treated with or without MG132 (10 μM, 6 hours), IP with Skp2 or ubiquitin antibody, and probed with DAB2IP or p27 antibody. The intensity of each band was measured using the Image-Pro Plus and normalized with Tubulin. (C) Both DAB2IP and Skp2 expression in PZ-HPV-7T cells treated with MG132 at indicated concentration were analyzed using western blot. (D) The ubiquitinated DAB2IP was determined in PZ-HPV-7T cells using *in vivo* ubiquitination assay. Ubi. ISG-15, ubiquitin-like interferon stimulated gene (*ISG*)-15, was used as a negative control. (E) PC3 cells were treated with or without MG132, IP with Skp2 antibody or control IgG, and immunoblotted with DAB2IP or Skp2 antibody. The intensity of each band was measured using the Image-Pro Plus and normalized with Actin. (F) Endogenous Skp2 protein expression was determined in 293wt and 293J cells using western blot. Actin was used for a loading control. (G) 293J cells were transfected with indicated plasmids and treated with 10 μM MG132 for 6 hours. Cell lysates were subjected to western blot probed with DAB2IP, Skp2, or p27 antibody, or *in vivo* ubiquitination assay. (H) 293wt cells were transfected with the indicated plasmids then both DAB2IP and Skp2 expression were determined with western blot.

We therefore decided to determine the impact of UPS on DAB2IP protein turnover. In the presence of proteasome inhibitor (MG132), DAB2IP protein elevated in PTN1A ρ(0) cells in a time-dependent manner (Fig. 2A). Using IP, we found that ubiquitinated DAB2IP form a complex with Skp2 and accumulated total and ubiquitinated DAB2IP was observed in MG132-treated PTN1A ρ(0) cells (Fig. 2B). Similarly, MG132 treatment resulted in the elevation of DAB2IP in PZ-HPV-7T cells (Fig. 2C). Also, ectopic ubiquitin expression resulted in increasing ubiquitinated DAB2IP but decreasing DAB2IP level in a dose-dependent manner (Fig. 2D). In PC3 cells, both elevated DAB2IP protein levels and DAB2IP-Skp2 complex were detected after treating with MG132 (Fig. 2E). Also, in 293J cells transiently transfected with DAB2IP expression vector, DAB2IP protein formed a complex with endogenous Skp2 in a dose-dependent manner (Fig. 2F). In addition, knocking down the endogenous Skp2 resulted in an elevation of DAB2IP protein and a reduction of ubiquitinated DAB2IP in a dose-dependent manner (Fig. 2G). Using constitutively active Skp2 [18, 19], reduced DAB2IP protein was detected in 293 wild-type (wt) cells (Fig.2H), Taken together, Skp2-mediated UPS plays an important role in regulating DAB2IP protein expression post-translationally in both immortalized normal prostate epithelial and cancer cells.

### N-terminal DAB2IP contains ubiquitination sites

**Figure 3:**
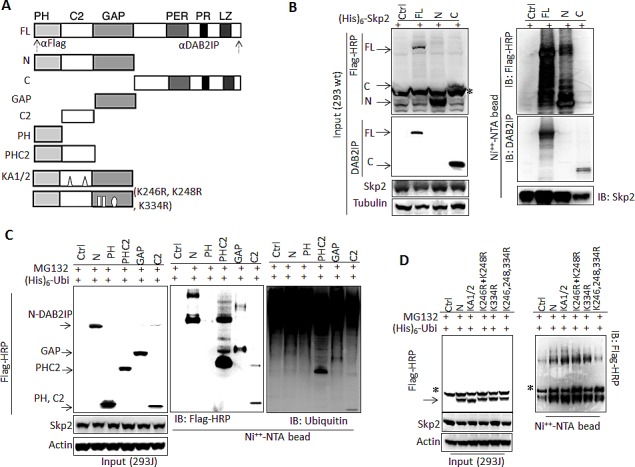
Determination of interactive domain in DAB2IP with Skp2 and its ubiquitination sites (A) Schematic representation of DAB2IP domain construct and the recognition sites of each antibody. (B-D) 293wt or 293J cells were transfected with a variety of DAB2IP domain constructs. Cell lysates were subjected to western blot or *in vivo* ubiquitination assay. * non-specific bands.

To map the ubiquitination site of DAB2IP protein, plasmids containing His-tagged Skp2 gene and different constructs of DAB2IP cDNA (Fig. 3A) were co-transfected into 293wt cells. Subsequently, His-tagged proteins were affinity-purified and analyzed by immunoblotting. Results (Fig. 3B) showed that both full-length and N-terminal DAB2IP protein could be ubiquitinated and form complexes with Skp2. Although Skp2 can bind to C-terminal DAB2IP, there is no ubiquitination site (Fig. 3B). Using different domains of N-terminal DAB2IPc DNA,construct data from *in vivo* ubiquitination assay further indicated that GAP, C2 and PHC2 domains, but not PH domain alone can be ubiquitinated and degraded (Fig. 3C). Similar findings were shown in the *in vivo* ubiquitination assay for FΔPH, FΔLZ, and GAPC fragments (Fig. S1). Furthermore, according to the predicted ubiquitination sites (http://ubpred.org/index.html) for N-terminalDAB2IP, there are three potential ubiquitination sites in GAP domain including K246, K248 and K334. Using site-directed mutagenesis, we found that the mutant containing all three sites significantly reduced DAB2IP ubiquitination (Fig. 3D).

### DAB2IP regulates Skp2 degradation through Akt signaling

**Figure 4:**
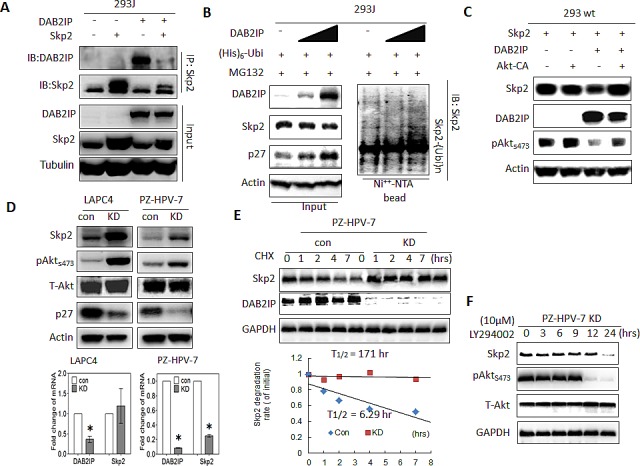
The effect of DAB2IP on Skp2 protein expression mediated through Akt (A) 293J cells were transfected with plasmids carrying *DAB2IP* or *Skp2* cDNA for 48 hours. Cell lysates were IP with Skp2 antibody and immunoblotted with DAB2IP or Skp2 antibody (B-C) 293 wt or 293J were transfected with the indicated plasmids. Cell lysates were subjected to western blot, or *in vivo* ubiquitination assays, respectively. (D) Cell lysates were harvested from control (Con) or DAB2IP knocked-down (KD) cells of LAPC4, PZ-HPV-7 then subjected to western blot and Actin was used as a loading control. Both DAB2IP and Skp2 mRNA expression in LAPC4 KD, PZ-HPV-7 KD and their control cells were determined using qRT-PCR assays. Data are represented as mean +/− SEM. (E) PZ-HPV-7 KD and con cells were treated with cycloheximde (15 μg/ml) at indicated time. Cell lysates were subjected to western blot. The expression of GAPDH was used as a loading control. Skp2 degradation rate was determined based on Skp2/GAPDH ratios at each time point of cycloheximide treatment. (F) PZ-HPV-7 KD cells were treated with 10 M LY294002 at indicated time. Cell lysates were subjected to western blot.

We noticed that DAB2IP was able to suppress Skp2 expression in 293J cells (Fig. 2F and 4A). Elevated expression of DAB2IP resulted in a reduction of Skp2 expression and an accumulation of ubiquitinated Skp2 in 293J cells (Fig. 4B). Further investigation in 293wt (Fig. 4C) showed that increased DAB2IP expression was able to decrease Skp2 protein levels and this reduction could be reversed in the presence of continuously active Akt (Akt-CA).

Moreover, by knocking down DAB2IP (KD), both LAPC4 and PZ-HPV-7 cells [24] exhibited increasing expression of Skp2 and phosphorylated Akt (pAkt) compared with the control cells (Con), the decreased p27 protein level revealed the degradation activity of Skp2 for its substrate (Fig. 4D). Increased Skp2 protein did not correlate with *Skp2* mRNA levels in DAB2IP KD cells (Fig. 4D), suggesting that the regulation of Skp2 protein is mediated by Akt at post-transcriptional level. Thus, we determined the half-life of Skp2 and the results showed that the half-life of Skp2 is longer in PZ-HPV-7 KD than its control cell (Fig. 4E). And also, in the presence of pAkt inhibitor LY294002 (10 μM), the half-life of Skp2 protein reduced significantly (Fig. 4F). Our results indicate that DAB2IP is able to facilitate Skp2 degradation by inhibiting Akt activity.

### The reciprocal regulation between DAB2IP and Skp2 is involved in the growth of prostatic epithelia both *in vitro* and *in vivo*

**Figure 5:**
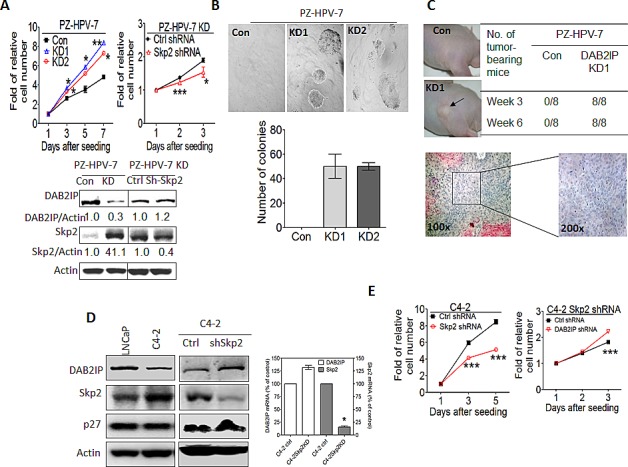
The effect of Skp2 on the tumor properties of prostatic cells from *in vitro* and *in vivo* (A) PZ-HPV-7 KD cells were transfected with control or Skp2 shRNA construct. DAB2IP and Skp2 expression were determined using western blot and actin was used as a loading control. One thousand cells/well were seeded using 96-well plate. *In vitro* cell growth was measured using MTT assays at the indicated time. Data are represented as mean +/− SEM. (B) PZ-HPV-7 KD cells and its control cells were plated on 24-well plate and the numbers of colony formation on soft agar were determined 2 weeks after plating. (C) PZ-HPV-7 KD cells and the control (1 × 10^6^ cells) were injected into the nude mice subcutaneously and tumor take were determined at the indicated time. Each tumor was excised for histological examination. (D) The DAB2IP, Skp2, and p27 protein expression in LNCaP, C4-2, C4-2 shSkp2 and its control cells were determined using western blot. Both DAB2IP and Skp2 mRNA expression in C4-2 Skp2 shRNA and its control cells were determined using qRT-PCR assays. Data are represented as mean ± SEM. (E) 1 × 10^3^ cells of C4-2 shSkp2 cells and its control were seeded at 96-well plate. *In vitro* cell growth was determined using MTT assays at the indicated time. Data are represented as mean +/− SEM. Twenty-four hours after the transfection of DAB2IP shRNA plasmids or control, C4-2 shSkp2 cells were seeded at 96-well plates then cell growth was determined using MTT assay. Data are represented as mean ± SEM.

To evaluate the impact of interaction between DAB2IP and Skp2 on cell growth, MTT assay and soft agar colony formation assay (CFA) were carried out by using immortalized normal prostate cell, PZ-HPV-7. As shown in Fig. 5A and B, PZ-HPV-7 KD cells displayed higher growth rate and numbers of cells formed in the colonies accompanied with increased expression of Skp2 protein levels. Knocking down Skp2 expression using transient transfection of Skp2 shRNA reversed the growth rate of PZ-HPV-7 KD cells (Fig. 5A). In addition, cells were implanted subcutaneously into nude mice and the potential of tumor growth of the cells were evaluated. DAB2IP KD cells formed tumors in 100% [18] of the experimental mice in an *in vivo* xenograft model (Fig. 5C). In general, PZ-HPV-7 KD acquired *in vitro* growth rate, anchorage independent growth, and *in vivo* tumorigenic potential. Reverse of cell growth rate by repressing Skp2 expression in PZ-HPV-7 KD suggests a regulatory role of the interaction between DAB2IP and Skp2 in cell proliferation. We further determined whether there is a similar role of interaction between DAB2IP and Skp2 in PCa cells. C4-2 cell line, an androgen-independent line derived from androgen-sensitive LNCaP [25], showed higher Skp2 expression and lower DAB2IP expression than LNCaP cells (Fig 5D). Knocking down the endogenous Skp2 in C4-2 cells resulted in an elevation of DAB2IP level accompanied with growth inhibition (Fig. 5D, E), in which the change of DAB2IP mRNA levels was not significant (Fig. 5D, right panel). Furthermore, knocking down DAB2IP mRNA in C4-2-Skp2 shRNA cells did restore the growth rate (Fig. 5E). Altogether, our data indicate that the Skp2- DAB2IP interaction can impact on PCa cell growth.

### The expressions of DAB2IP and Skp2 in human PCa specimens

**Figure 6:**
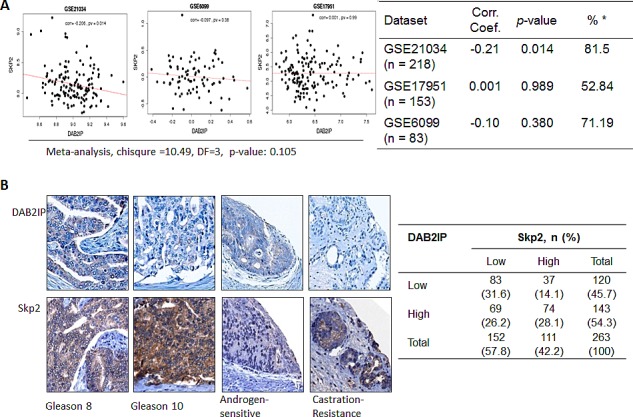
The expression of DAB2IP and Skp2 in human PCa specimens (A) Three datasets of cDNA array from PCa patients were analyzed for the correlation between *DAB2IP* and *Skp2* mRNA expression. (B) Two tissue microarrays of PCa tissues were immunostained with DAB2IP Skp2 antibody. Right panel: the representative images were photographed and displayed. Left panel: the summary Table with sample number and percentage in parenthesis.

Owing to the inverse correlation of DAB2IP and Skp2 proteins, which was not due to transcriptional regulation, observed in cell lines, we would like to find out whether the phenomenon can be seen in clinical specimens. We explored three different datasets of cDNA arrays (GSE21034, GSE6099, and GSE17951) of human prostate cancer patients to see whether similar feature can be found. The correlation co-efficiencies were −0.21, 0.001, and −0.10, respectively. Only GSE21034 dataset showed a significantly inverse relationship between *DAB2IP* and *Skp2* mRNA expression (*p* = 0.014). Overall, there was no significant correlation between DAB2IP and Skp2 using a meta-analysis method (chi-square =10.49, DF=3, p = 0.105) (Fig. 6A). Additionally, we probed both DAB2IP and Skp2 proteins in two tissue microarrays containing 263 PCa specimens using immunohistochemical staining (IHC). Among them, 69 (26.2%) or 37 (14.1%) PCa specimens exhibited DAB2IP^high^ -Skp2^low^ or DAB2IP^low^-Skp2^high^ pattern, respectively (Fig. 6B). Although statistically there is no correlation between the expressions of these two proteins in PCa specimens, an inverse correlation was still observed in approximate 40% of the PCa specimens.

## DISCUSSION

DAB2IP is known as a tumor suppressor in several cancers, such as breast, lung and hepatocellular carcinoma [26-28]. In addition, genome-wide association studies also indicate that single nucleotide polymorphism of DAB2IP gene is associated with not only the risk of aggressive PCa and other non-malignant diseases such as abdominal aortal aneurysm and cardiovascular diseases [29, 30]. In general, loss of DAB2IP in cancer cells is due to its epigenetic silencing [5, 6, 26-28]. However, in this study, we unveil additional mechanism leading to the loss of DAB2IP protein that is regulated by Skp2-mediated UPS. Interestingly, DAB2IP is also able to regulate Skp2 protein stability through Akt-mediated pathway [18, 31]. Most importantly, the reciprocal regulation between these two proteins plays an important role in influencing tumor behaviors of PCa.

In our results, although N- or C-terminal domain of DAB2IP protein can interact with Skp2, several potential ubiquitination sites are found in the C2 and GAP domain of the N-terminal. The lysine-rich clusters found in the C2 domain that can bind to Ask1, PP2A, and GSK-3βleading to enhance cell apoptosis or prevent epithelial-to-mesenchymal transition [8, 24, 32] appear not the ubiquitination sites for Skp2. In contrast, within the GAP domain, K246, K248 and K334 are key sites for Skp2-eliicted ubiquitination. On the other hand, Skp2 recognizes substrate(s) for ubiquitylation usually through the phosphorylated consensus sequence(s) rather than recognizing a degron [33-35]. For instance, the phosphorylated Thr187 of p27(Kip1) binds to Skp2 through Cks1-phosphate binding site [34]. Nevertheless, the consensus sequence for phosphorylation in Skp2 substrates and whether it is essential for initiating the ubiquitination are still not fully understood. Similarly, the requirement of DAB2IP phosphorylation in Skp2 recognition needs further study.

Interestingly, DAB2IP can also regulate Skp2 protein stability in normal or benign cells. It is known that the regulation of Skp2 degradation is complex and involves multiple mechanisms. Skp2 gene expression can be regulated by p53 and NF-κB through Akt-GSK-3β pathway [21]. Also, TCF4 and β-catenin can regulate Skp2 gene expression through the binding of TCF/LEF1 to Skp2 promoter [20]. Besides the regulation at gene expression level, Skp2 can be degraded via auto-ubiquitination in Cul1-dependent [36], or Cdh1 dependent manners. Also, p107 has been reported to promote Skp2 degradation independent of either Cul1 or Cdh1 [37]. In addition, Akt mediated phosphorylation stabilized Skp2 by evading from APC/Cdh1-mediated proteasomal degradation [31, 38]. Since DAB2IP can function as a signalosome platform for coordinating protein-protein interaction from various signaling pathways including Ask1-JNK [8, 32], PIK3-Akt [32], PP2A-β catenin [24], and NF-κB [5], it is likely that DAB2IP modulate Skp2 through these pathways, especially through inhibiting Akt activity. However, we can't completely rule out any other pathways also involved in this regulation.

Several previous studies using PCa specimens clearly indicate the association of Skp2, as a potential oncoprotein, with disease progression. De Marzo *et al*. and Arbini *et al*. reported that nuclear staining of Skp2 in PCa specimens is associated with more aggressive behavior [39, 40]. Other studies indicated that the cytoplasmic Skp2 protein exhibits E3 ubiquitin ligase activity and correlates with disease progression [18, 19]. Drobnjak *et al* reported that Skp2 staining in African-American, a population known to have the highest risk and more aggressive type of this cancer, PCa specimens is mainly cytoplasmic [41]. Furthermore, the accumulation of cytoplasmic Skp2 due to Akt-elicited Skp2 phosphorylation at serine 72 was associated with tumor cells expressing elevated Akt or reduced PTEN [19, 31]. In this study, about 40% of the PCa specimens showed an inverse correlation either DAB2IP^low^/Skp2^high^ (14.1%) or DAB2IP^high^/Skp2^low^ (26.2%). Taken together, Skp2 is a potent oncoprotein in subset of PCa patients.

In summary, we demonstrated a reciprocal regulation between DAB2IP, a tumor suppressor, and Skp2, an oncogenic protein, in normal prostatic epithelia and PCa cells, which represents paradigm shift of signalosome pattern in normal cell to malignant tumor. Based on these findings, it provides new therapeutic strategy for targeting Skp2 as a targeted therapy in PCa patients.

## MATERIALS AND METHODS

### Plasmid constructs

Various expression plasmids for DAB2IP: F-, C-, N-, PH, PHC2, KA1/2, FΔPH, FΔLZ and DAB2IP shRNA were described previously [8, 24, 42, 43]. Additional expression plasmids: C2, GAP, GAPC from N-DAB2IP; FΔPH from F-DAB2IP; 3 mutants from N-DAB2IP (i.e., K246R/K248R, K334R, and K246R/K248R/K334R) using site-directed mutagenesis kits (QuikChange®, Stratagene). Skp2shRNA (sc-36499-SH) and its control plasmid were purchased from Santa Cruz Biotechnology. Skp2 cDNA and its derivative mutants (S72A, S72D, S64A, S64D) were kindly gifted from Dr. Hui-Kuan Lin (MD Anderson Cancer Center, Houston, TX) [19]. The plasmids pcDNA3.1-ubiquitin, and pcDNA3.1- ubiquitin ISG15 were obtained from Dr. Dimitris Xirodimas (University of Dundee, Scotland, UK). The plasmid carrying Akt-CA cDNA was provided by Dr. David Boothman (UT Southwestern Medical Center, Dallas, TX).

### Cell culture, Antibodies, Reagents, and plasmids transfection

PNT1A, PC3, LNCaP, PZ-HPV-7, C4-2, LAPC4, 293 and their sublines were maintained as described previously [22-24, 32]. Anti-DAB2IP polyclonal antibody was used for western blot analysis and IHC as described previously [24, 32]. Anti-FLAG-HRP (M2) was obtained from Sigma (St. Louis, MO). Anti-Skp2 (sc74477), anti-Ubiquitin (sc271289), anti-Tubulin (32239), anti-Akt 123 (H36, sc8312), and anti-GAPDH (sc16674) were purchased from Santa Cruz Biotechnology (Santa Cruz, CA). Anti-phospho-Akt (Ser473) polyclonal antibody (#9271) was from Cell signaling Technology (Danvers, MA). Anti-p21 (6B6) and Anti-p27 were obtained from BD Pharmingen (Sparks, MD). Anti-Skp2 (2C8D9) was from Zymed (South San Francisco, CA) Proteasome inhibitor MG132 was purchased from Calbiochem (Gibbstown, NJ), cycloheximide and 2-(4-morpholinyl)-8-phenyl-chromone (LY294002) were also purchased from Sigma. For cDNA transfection, cells were seeded in plates with 70-80% confluence before transfection. The transfection was carried out using Lipofectamine LTX with Plus™ reagent (Invitrogen, Carlsbad, CA) or polyethylenimine (PEI, Polysciences Inc., Warrington, PA) according to the manufacturer's instructions.

### qRT-PCR analysis

The total RNA was extracted with RNeasy mini kit (Qiagen, Valencia, CA) treated with RNase-free DNase I (Qiagen) and subjected to a cDNA synthesis kit (Bio-Rad, Hercules CA). The cDNA was further amplified in a 25 ml quantitative PCR reaction mixture containing 12.5μg of iQ^™^ SYBGREEN Supermix® (Bio-Rad) and the studied primers using an iCycleriQ machine (Bio-Rad). The sequences of primers forDAB2IP are 5'-TGGACGATGTGCTCTATGCC-3' (forward) and 5'-GGATGGTGATGGTTTGGTAG-3' (reverse). Primers for Skp2 are 5'-AGCCCGACAGTGAGAACATC-3' (forward) and 5'-GAAGGGAGTCCCATGAAACA -3' (reverse). Primers for 18S RNA are 5'-GGAATTGACGGAAGGGCACCACC-3' (forward) and 5'-GTGCAGCCCCGGACATCTAAGG-3' (reverse). The PCR amplification protocol was 95 °C (3 minutes), 36 cycles of amplification cycle (95 °C [30 second], 55 °C [30 second], and 72 °C [1 minute]). All the data were done in duplicates and were repeated at least twice. The relative level of *DAB2IP* or *Skp2* mRNA from each sample was calculated by normalizing with *18S*cDNA.

### Western blot assay

For western blot analysis, cells were washed twice with cold PBS first and lysed in 1.5 mL of cold RIPA buffer [44]mixed with fresh complete protease inhibitor cocktail (Roche, Indianapolis, IN) for 20 minutes on ice. After sonication with a microtipped sonifier at setting 3 for 20 seconds to reduce viscosity and centrifugation, cell lysates were subjected to western blot analysis.

### Immunoprecipitation (IP)

Cell lysates lysed with RIPA buffer were further subjected to IP. In brief, anti-Skp2, anti-ubiquitin, or their control antibodies were incubated with Dynabead (Invitrogen) first for 15 minutes at room temperature, and mixed with the indicated cell lysates for 45 minutes. The eluted fraction was further immunoblotted with DAB2IP, Skp2, or other antibodies indicated in each figure.

### *In vivo* Ubiquitination assay

This assay was modified from Treier et al [45] and McMahon et al [46]. The input fraction was prepared using RIPA buffer. His-tagged protein was pulled down using Dynabead^®^ His-Tag Isolation & Pulldown (Invitrogen) or MagneHis^™^ Protein Purification System (Promega, Madison, WI). Briefly, HEK293 cells (1 × 10^6^) seeded at a 10-cm dish were transfected with pcDNA3.1 His-Ubiquitin, or His-Skp2 vectors along with the indicated plasmids. Approximately 36 h after transfection, cells were treated with 10 μM MG132 for 6 hours. Then, cell pellets were harvested and equally aliquoted into three 1.5 cm eppendorf tubes for the input, pull-down, and backup. The backup tube was immediately stored at −80 °C freezer. The input fraction was prepared using the RIPA buffer described as above. His-tagged protein was pulled down using Dynabead^®^ His-Tag Isolation & Pulldown (Invitrogen) or MagneHis™ Protein Purification System (Promega). Briefly, the cell suspension was lysed by adding 1.0 ml of buffer A (6 M guanidinium-HCI, 0.1 M Na_2_HPO_4_/NaH2PO_4_ pH 8.0 supplemented with 5 mM imidazole). The cell lysate was mixed with 50 μl of His-Tag magnetic beads and sonicated, then the mixture was incubated at room temperature for 2 hours and overnight at 4 °C. Thereafter, the beads were washed sequentially with buffer A supplemented with 10 mM 2-mercaptoethanol (2-ME), buffer B (8 M urea, 10 mMTris, 0.1 MNa_2_HPO_4_/NaH2PO_4_, pH 8.0) supplemented with 10 mM 2-ME, buffer C (8 M urea, 10 mMTris, 0.1 M Na_2_HPO_4_/NaH2PO_4_, pH 6.3) supplemented with 10 mM 2-ME and 0.2% (v/v) Triton X-100, and finally, buffer C supplemented with 10 mM 2-ME and 0.1% (v/v) Triton X-100. Bound material was eluted from the beads by suspension in 75 μL of modified Laemmli sample buffer (20 mMTris-Cl, pH 6.8, 10% [v/v] glycerol, 0.8% [w/v] SDS, 0.1% [w/v] bromphenol blue, 720 mM 2-ME, and 500 mM imidazole) followed by the incubation at 70°C water bath for 10 minutes. The eluted samples were collected and referred to as the “pull-down” fraction. Both of the input and pull-down fraction were subjected to SDS-PAGE and western blot analyses.

### MTT assay and Soft agar colony formation assay

For cell growth assay, 1 × 10^3^ cells per well were seeded in 96-well plates for the indicated time. Cell growth rate was calculated using 3-(4,5-dimethylthiazol-2-yl)-2,5-diphenyltetrazolium bromide (MTT) assay (Roche). The relative cell number was calculated by normalizing with Day 1 (=1).

For soft agar colony formation assay, 1 × 10^3^ cells/well were plated on agar in the 24-well plates according to Sato et al [47].Two weeks later, the plates were fixed with 4% paraformaldehyde and stained in crystal violet solution. The number of colony was counted.

### Xenograft formation and Histology examination

PZ-HPV-7 KD and its control (1 × 10^6^) cells were injected into nude mice subcutaneously. The tumor incidence was recorded every other day until 6 weeks after inoculation. All the tumors were excised for histological examination using H&E staining.

### IHC Staining

Two serial sections of two tissue microarrays containing human prostate tumor tissues were subjected to Ventana autostainer model Discover XT ™ (Ventana Medical System, Tuscan, AZ). The primary antibodies were anti-DAB2IP [23, 24]and anti-Skp2 (2C8D9) from Zymed, San Francisco, CA. Two pathologists assessed and scored the immunostaining independently and reached a final consensus for any inconsistent scoring. Briefly, values on a four-point scale were assigned to each specimen. The intensity score was assigned, which represented the average intensity of positive cells (0, none; 1, weak or questionably present stain; 2, intermediate intensity in a minority of cells; and 3, strong intensity in a majority of cells). High expression was defined as score higher than average, and low expression was defined as score lower than average.

### Microarray database analysis

Three microarray data sets of PCa were obtained from the NCBI Gene Expression Omnibus (GEO): GSE21034 (n = 218) [48], GSE6099 (n = 83) [49], and GSE17951 (n = 153) [50]. Using quantile normalization, the Spearman correlation coefficient was calculated between *DAB2IP, Skp2* and individual genes and ranked in individual data sets as described previously [51]. All data were analyzed by using GraphPad Prism 5 (GraphPad, Inc., La Jolla, CA) and SPSS13.0 software package (SPSS Inc., Chicago, IL). The *p*<0.05 was considered as significant.

## SUPPLEMENTARY INFORMATION AND FIGURE


