# Supplementary material for: The role of homeostatic regulation between tumor suppressor DAB2IP and oncogenic Skp2 in prostate cancer growth
Source: Oncotarget. 2014 Jul 17;5(15):6425–36. doi: 10.18632/oncotarget.2228 (PMC4171641; doi:10.18632/oncotarget.2228)
Supplement: Supplementary file 1 [file oncotarget-05-6425-s001.pdf]

The role of homeostatic regulation between tumor suppressor DAB2IP and oncogenic Skp2 in prostate cancer growth

Supplementary Information

The sequences of primers for DAB2IP are 5'-TGGACGATGTGCTCTATGCC-3’ (forward) and 5'-GGATGGTGATGGTTTGGTAG-3' (reverse). Primers for Skp2 are 5’-AGCCCGACAGTGAGAACATC-3’ (forward) and 5’-GAAGGGAGTCCCATGAAACA -3’ (reverse). Primers for 18S RNA are 5'-GGAATTGACGGAAGGGCACCACC-3' (forward) and 5'-GTGCAGCCCCGGACATCTAAGG-3’ (reverse).

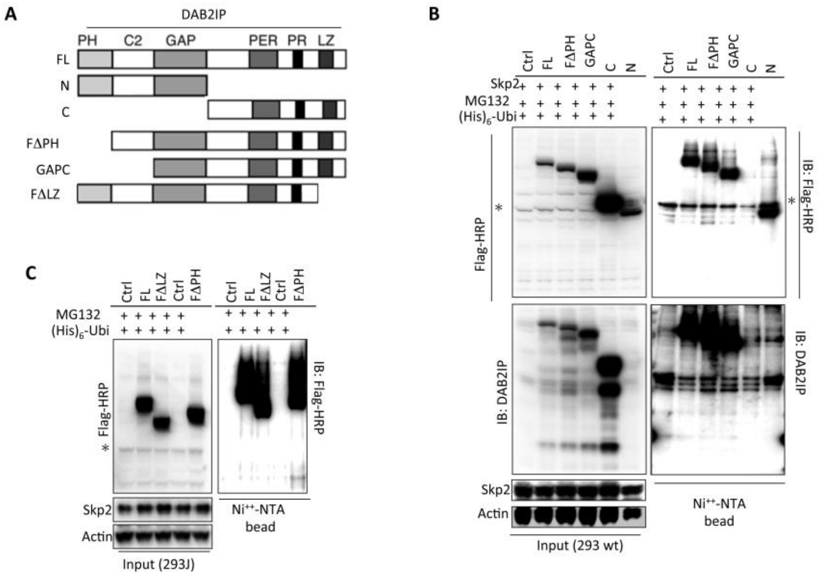

Sup Fig: (A) Schematic representation of DAB2IP domain construct. (B, C) 293 wt or 293J cells were transfected with the indicated DAB2IP domain constructs. Cell lysates were subjected to western blot, or *in vivo* ubiquitination assay.
